# Trajectories of urinary incontinence in childhood and bladder and bowel symptoms in adolescence: prospective cohort study

**DOI:** 10.1136/bmjopen-2016-014238

**Published:** 2017-03-10

**Authors:** Jon Heron, Mariusz T Grzeda, Alexander von Gontard, Anne Wright, Carol Joinson

**Affiliations:** 1 School of Social and Community Medicine, University of Bristol, Bristol, UK; 2 Department of Child and Adolescent Psychiatry, Saarland University Hospital, Homburg, Germany; 3 Evelina London Children's Hospital, St Thomas’ Hospital, London, UK

## Abstract

**Objectives:**

To identify different patterns (trajectories) of childhood urinary incontinence and examine which patterns are associated with bladder and bowel symptoms in adolescence.

**Design:**

Prospective cohort study.

**Setting:**

General community.

**Participants:**

The starting sample included 8751 children (4507 men and 4244 women) with parent-reported data on frequency of bedwetting and daytime wetting for at least three of five time points (4½, 5½, 6½, 7½ and 9½ years—hereafter referred to as 4–9 years). Study children provided data on a range of bladder and bowel symptoms at age 14 (data available for 5899 participants).

**Outcome measures:**

Self-reported bladder and bowel symptoms at 14 years including daytime wetting, bedwetting, nocturia, urgency, frequent urination, low voided volume, voiding postponement, passing hard stools and low stool frequency.

**Results:**

We extracted 5 trajectories of urinary incontinence from 4 to 9 years using longitudinal latent class analysis: (1) normative development of daytime and night-time bladder control (63.0% of the sample), (2) delayed attainment of bladder control (8.6%), (3) bedwetting alone (no daytime wetting) (15.6%), (4) daytime wetting alone (no bedwetting) (5.8%) and (5) persistent wetting (bedwetting with daytime wetting to age 9) (7.0%). The persistent wetting class generally showed the strongest associations with the adolescent bladder and bowel symptoms: OR for bedwetting at 14 years=23.5, 95% CI (15.1 to 36.5), daytime wetting (6.98 (4.50 to 10.8)), nocturia (2.39 (1.79 to 3.20)), urgency (2.10 (1.44 to 3.07)) and passing hard stools (2.64 (1.63 to 4.27)) (reference category=normative development). The association with adolescent bedwetting was weaker for children with bedwetting alone (3.69 (2.21 to 6.17)).

**Conclusions:**

Trajectories of childhood urinary incontinence are differentially associated with adolescent bladder and bowel symptoms. Children exhibiting persistent bedwetting with daytime wetting had the poorest outcomes in adolescence.

**Strengths and limitations of this study**

- A major strength of the study is the availability of repeated measures of bedwetting and daytime wetting across childhood in a large, representative cohort.
- The study is the first to model parallel trajectories of bedwetting and daytime wetting across childhood.
- Adolescent bladder and bowel symptoms were based on self-report rather than clinical diagnosis.
- Information about lower urinary tract symptoms, soiling or constipation, and organic causes of incontinence was not included in the trajectories.
- We did not consider whether treatment for urinary incontinence might have impacted on the findings.

## Introduction

Urinary incontinence is common in childhood^1^ ^2^ and, if poorly managed, can seriously undermine a child's quality of life and self-esteem.^3^ Children who attend continence clinics have high rates of comorbid emotional distress, with 20–40% meeting diagnostic criteria for psychiatric disorders.^4^ Despite the prevalence and impacts, little is known about the prognosis of childhood incontinence. Parents and clinicians often adopt a ‘wait and see’ approach to childhood incontinence, due to the common belief that it resolves with age.^5^ A significant proportion of children, however, continue to suffer from persistent incontinence into adolescence^1^ ^6^ ^7^ and adulthood.^8^ Increased understanding of the prognosis of childhood incontinence is needed to improve identification of those who should be prioritised for specialist services to help prevent chronic incontinence and secondary impacts.

There is evidence that adolescents experience more severe incontinence than children.^1^ A cross-sectional study of 5–19-year-olds found a greater proportion of frequent bedwetting (≥3 wet nights/week), accompanied by daytime wetting and other lower urinary tract symptoms (LUTS), in older (11–19 years) compared with younger children (5–10 years).^1^ The authors argue that this provides evidence that frequent bedwetting with daytime LUTS, referred to as non-monosymptomatic nocturnal enuresis (NMNE), is less likely to resolve with age. NMNE can often be attributed to overactive bladder (OAB, syndrome characterised by urge symptoms, increased urination frequency and small voided volumes)^9^ and is distinguished from monosymptomatic NE (MNE, bedwetting without daytime symptoms),^10^ which is often attributed to nocturnal polyuria and a deficit in the basic inhibitory function of the brainstem leading to a lack of inhibition of the micturition reflex during sleep.^11^ A prospective cohort study found that childhood urinary incontinence (UI; wetting in the day or several nights a week at age 6) was associated with severe incontinence and urge symptoms in women at age 48, while occasional bedwetting was not linked to adult incontinence.^12^ There are also associations reported between retrospective reports of childhood UI and adult UI and LUTS,^13–16^ childhood bedwetting and adult nocturia,^17^ and childhood bladder and bowel symptoms and bedwetting in adolescents and adults.^18^ UI is known to be linked to bowel habits (eg, low stool frequency and passage of hard stools),^19^ but no prospective studies have examined whether childhood incontinence is related to bowel symptoms in adolescence. All previous studies have examined only the presence or absence of incontinence and/or bladder and bowel symptoms in childhood and the association with subsequent daytime wetting, bedwetting and/or LUTS. None have taken into account the heterogeneity in developmental trajectories of incontinence during childhood. There is evidence for distinct patterns of UI characterised by delayed attainment of bladder control, persistent wetting and relapses.^20–23^ No studies have examined whether different patterns of UI in childhood are differentially associated with adolescent outcomes. The current study uses longitudinal data from a large UK cohort to identify subgroups of children who differ in patterns of daytime wetting and bedwetting during childhood. The aims are to study the overlap between patterns of daytime wetting and bedwetting in childhood (4–9 years) and to examine whether these patterns are differentially associated with bladder and bowel symptoms in adolescence (14 years).

## Methods

The sample comprised participants from the Avon Longitudinal Study of Parents and Children (ALSPAC). Detailed information about ALSPAC is available on the study website (http://www.bristol.ac.uk/alspac), which includes a fully searchable dictionary of available data (http://www.bris.ac.uk/alspac/researchers/data-access/data-dictionary). Pregnant women residents in the former Avon Health Authority in south-west England, having an estimated date of delivery between 1 April 91 and 31 December 92, were invited to take part, resulting in a cohort of 14 541 pregnancies and 13 973 singletons/twins (7217 boys and 6756 girls) alive at 12 months.^24^

### Exposures: daytime wetting and bedwetting during childhood

Postal questionnaires were sent out to parents when the study children were aged 4½, 5½, 6½, 7½ and 9½ years (hereafter referred to as 4–9 years). Over 90% of questionnaires were returned within 1–2 months of the target age. Parents were asked ‘How often usually does your child wet him/herself during the day?’ and ‘during the night?’ and for both questions were given the response options ‘Never’; ‘Occasional accidents but less than once a week’; ‘About once a week’; ‘2–5 times a week’; ‘Nearly every day; and ‘More than once a day’.

### Outcomes: bladder and bowel symptoms in adolescence

A self-report postal questionnaire was sent out to study children when they were 13 years 10 months (IQR: 13 years 10 months—13 years 11 months, hereafter referred to as 14 years; over 90% of participants returned the questionnaire within 3 months of the target age). Of the 8751 samples, 5899 (67.4%) returned the 14-year questionnaire. The questionnaire included questions about the frequency of incontinence (daytime wetting and bedwetting), LUTS (nocturia, urgency, frequent urination, low voided volume and voiding postponement) and bowel symptoms (passing hard stools and stool frequency) over the last 2 weeks. Table 1 provides the exact questions used to derive these outcomes.

**Table 1:** Prevalence and gender differences in bladder and bowel symptoms at age 14 years

				Prevalence of outcome			
Outcomes	Responses coded as 0	Responses coded as 1	N	Whole sample (%)	Male (%)	Female (%)	p Value
How often do the following happen to you?							
* Daytime wetting* Wet yourself during the day?	‘never’	‘less than once a week’, ‘about once a week’, ‘2–5 times a week’, ‘nearly every day’ and ‘more than once a day’	5803	2.9	1.3	4.2	<0.001
* Bedwetting* Wet the bed at night?	‘never’	‘less than once a week’, ‘about once a week’, ‘2–5 times a week’, ‘nearly every day’ and ‘more than once a day’	5805	2.5	2.9	2.2	0.08
Over the last 2 weeks, how often have you:							
* Nocturia* Woken up to go for a wee?	‘never’/‘a few times’	‘quite often’/‘a lot’	5779	9.2	8.7	9.7	0.17
* Urgency* Had a sudden feeling you need a wee and had to dash to the toilet?	‘never’/‘a few times’	‘quite often’/‘a lot’	5806	4.8	4.3	5.2	0.10
* Frequent urination* Had to go to the toilet for a wee more than 7 times a day?	‘never’/‘a few times’	‘quite often’/‘a lot’	5792	2.7	2.2	3.1	0.05
* Low voided volume* Passed only a small amount when you went for a wee?	‘never’/‘a few times’	‘quite often’/‘a lot’	5768	4.4	3.6	5.1	0.004
* Voiding postponement* Avoided going for a wee until the last moment because you were concentrating on other activities?	‘never’/‘a few times’	‘quite often’/‘a lot’	5787	13.7	12.8	14.5	0.06
* Passing hard stools* Had hard stools (poos) that were difficult to pass?	‘never’/‘a few times’	‘quite often’/‘a lot’	5766	2.7	2.7	2.7	0.84
* Low stool frequency* How often do you usually pass a stool (do a poo)?	‘3 or more times a day’, ‘twice a day’, ‘once a day’ and ‘every other day’	‘every third day’ and ‘less often than every 3rd day’	5696	9.2	7.6	10.6	<0.001

There was overlap between those reporting daytime and bedwetting; 36 (0.6%) reported both, 130 (2.2%) reported daytime wetting alone and 102 (1.8%) reported bedwetting alone (data not shown).

### Estimation of the parallel trajectory model

We previously used longitudinal latent class analysis (LLCA) to derive separate models of daytime wetting and bedwetting in childhood.^21^ ^23^ In this earlier work, we showed that patterns of daytime wetting at 4–9 years could be adequately explained by a four-class solution and patterns of bedwetting could be explained by five classes (see online supplementary material). *Separate* latent class models for daytime wetting and bedwetting ignore the comorbidity between these continence problems. Therefore, in the current study, we derived a ‘parallel LLCA’ (using in Mplus V.7.11, Muthén & Muthén) in order to describe the repeated bivariate data of daytime wetting and bedwetting in tandem. We then examined the association between the latent classes and bladder and bowel symptoms in adolescence using logistic regression. Full details of the analysis employed to extract the parallel latent classes are provided in online supplementary material and in tables S1 and S2 and figures S1–S4.

10.1136/bmjopen-2016-014238.supp1 supplementary material

10.1136/bmjopen-2016-014238.supp2 supplementary tables and figures

## Results

We focused on the sample of children (n=8751; 4507 men and 4244 women) with incontinence data for at least three of the five time points. We have previously shown our findings to be robust to the sample chosen,^23^ and there is little benefit to including those providing a small amount of childhood data since few provide follow-up data at age 14. Owing to a minor degree of item non-response, available data for each adolescent outcome at age 14 ranged from 5806 (urgency) to 5696 (low stool frequency) (see table 1). There were some observable differences between participants who provided data on adolescent bladder and bowel symptoms at 14 years and, therefore, were included in the analysis (n=5899) and those who did not provide data at 14 years and were excluded from the analysis (n=2852) (table 2). Men were more likely to be excluded from the analysis at 14 years. There were also differences in the socioeconomic variables, with children from less advantaged families and with less educated mothers being more likely to be excluded from the analysis at 14 years. It is notable, however, that the included and excluded samples had very similar proportions of children with bedwetting and daytime wetting at age 7.

**Table 2:** Characteristics of participants who did not provide data on bladder and bowel symptoms at 14 years (n=2852 excluded) compared with those who did (n=5899 included)

	Excluded at 14 years (n=2852)	Included at 14 years (n=5899)
Gender		
* *Female	38.5%	53.4%
* *Male	61.5%	46.6%
n	2852	5899
Social class*		
* *Non-manual (professional, managerial and skilled professions)	75.6%	82.3%
* *Manual (partly or unskilled occupations)	24.4%	17.8%
N	1195	2890
Home ownership*		
* *Owner/occupier	76.3%	86.1%
* *Rented accommodation	23.7%	13.9%
n	2537	5556
Car access*		
* *Yes	87.9%	94.4%
* *No	12.1%	5.6%
n	2475	5553
Maternal education*		
* *A-level or above	31.0%	45.4%
* *O-level	36.7%	35.0%
* *Certificate of secondary school/vocational/none	32.3%	19.6%
n	2737	5782
Bedwetting at 7 years		
* *Yes	16.7%	14.75%
* *No	83.3%	85.25%
n	2238	5513
Daytime wetting at 7 years		
* *Yes	7.8%	7.82%
* *No	92.2%	92.18%
n	2241	5514

*These variables were derived from responses to a questionnaire completed by mothers during the antenatal period.

supplementary table S2 summarises the probability of missing data on the 14-year outcomes across each latent class. To diagnose the impact of missing data on the results of the analysis investigating associations between class membership and bladder and bowel symptoms in adolescence, we used a series of bivariate logistic regressions predicting missing values on outcome variables (measured at 14 years) from class membership (see online supplementary table S2). This result suggested that the missing data mechanism was completely at random. Thus, the impact of missing values on the results seemed to be minimal; therefore, we decided that there was not a strong argument for employment of multiple imputation methods to impute missing data.

### Parallel model of bedwetting and daytime wetting

A solution comprising four classes of daytime wetting and five classes of bedwetting provided an adequate fit to the longitudinal bivariate data and explained the longitudinal heterogeneity and the cross-sectional association between daytime wetting and bedwetting at each time point. Table 3 presents the association between classes of daytime wetting and bedwetting during the period 4–9 years, which includes the ORs constructed for the parallel distribution of daytime wetting and bedwetting latent classes. A membership of the normative class on both dimensions was used as the reference category in our analysis. There is a tendency for a delay in the attainment of daytime bladder control to be associated with a delay in the attainment of night-time bladder control and vice versa. There is also a strong association between persistent classes for daytime wetting and bedwetting.

**Table 3:** Associations between latent classes of daytime wetting and bedwetting

		Bedwetting classes			
		Infrequent delayed	Frequent delayed	Infrequent persistent	Frequent persistent
Daytime wetting classes	Delayed	18.4 (15.4 to 22.0)	12.2 (8.56 to 17.5)	14.9 (11.9 to 18.6)	20.8 (15.0 to 28.9)
	Relapse	1.71 (1.24 to 2.38)	2.1 (1.03 to 4.29)	8.22 (6.3 to 10.7)	6.9 (4.31 to 11.0)
	Persistent	5.57 (4.18 to 7.42)	9.3 (5.70 to 15.3)	16.6 (12.6 to 21.9)	43.1 (30.7 to 60.7)

The table shows ORs and 95% CIs for the associations between bedwetting classes (in columns) and daytime wetting classes (in rows). The *Normative* class on both dimensions was the reference category. The ORs can be interpreted as follows: for example, the OR of 18.4 in the top left-hand cell indicates that membership of the *infrequent delayed* bedwetting class increases the odds of membership of the delayed class for daytime wetting, compared with the normative class, by over 18-fold.

The four-by-five-class solution represented 20 separate subgroups corresponding to each combination of daytime wetting and bedwetting. It is not practical to examine the risk of each adolescent outcome within each of these groups. Consequently, we collapsed these 20 groups into five distinct, clinically relevant classes through reaching a consensus with clinical experts (see table 4). Full details of the results of the statistical analysis used to extract the latent classes are provided in online supplementary tables S1 and S2 and figures S1–S4.

**Table 4:** Combining the four-by-five-class solution into the five parallel classes of daytime wetting and bedwetting (n=8751)

		Bedwetting classes				
		Normative (%)	Infrequent delayed (%)	Frequent delayed (%)	Infrequent persistent (%)	Frequent persistent (%)
Daytime wetting classes	Normative	63.0	9.0	1.3	4.1	1.2
	Delayed	2.3	6.1	0.6	2.2	0.9
	Relapsing	2.2	0.5	0.1	1.2	0.3
	Persistent	1.3	1.0	0.3	1.4	1.0

	Class name	Description	Number in class (%)	% of males and females
	Normative development of bladder control	Normative classes for daytime wetting and bedwetting	5513 (63.0%)	47.5 and 52.5
	Delayed attainment of bladder control	Infrequent and frequent delayed bedwetting classes combined with non-normative daytime wetting classes	752 (8.6%)	52.9 and 47.1
	Bedwetting alone (no daytime wetting)	Non-normative bedwetting classes combined with normative daytime wetting class	1365 (15.6%)	68.4 and 31.6
	Daytime wetting alone (no bedwetting)	Non-normative daytime wetting classes combined with the normative bedwetting class	508 (5.8%)	33.5 and 66.5
	Persistent wetting (persistent bedwetting with daytime wetting)	Infrequent persistent and frequent persistent bedwetting classes combined with non-normative daytime wetting classes	613 (7.0%)	63.0 and 37.0

Data shown in the table are class proportions for the latent class patterns based on the estimated model. These results are for the sample of children (n=8751) with incontinence data for at least three of the five time points.

### Association between the parallel latent classes of daytime wetting and bedwetting at 4–9 years and the adolescent bladder and bowel symptoms

**Figure 1:**
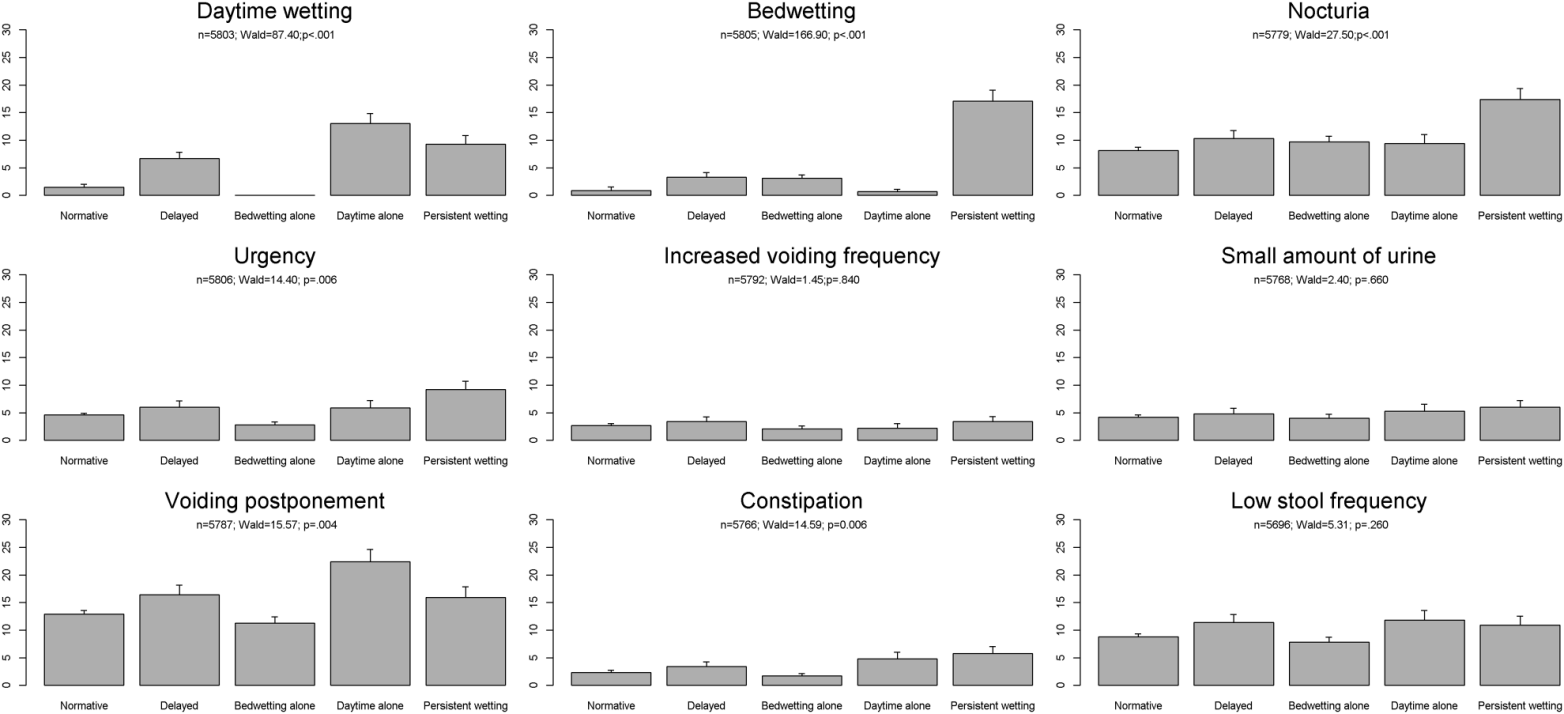
Associations between urinary incontinence at 4–9 years and bladder and bowel symptoms at 14 years. The prevalence of each adolescent bladder and bowel symptom in each of the five parallel latent classes of daytime wetting and bedwetting is shown. p Values are derived from the Wald test.

Figure 1 shows the associations between the parallel latent classes of daytime wetting and bedwetting and the adolescent outcomes. Table 5 provides the ORs and 95% CIs for these associations.

**Table 5:** ORs and 95% CIs for the association between the latent classes of daytime wetting and bedwetting at 4–9 years and bladder and bowel symptoms at 14 years

Outcome variable	Normative*	Delayed†	Bedwetting alone‡	Daytime wetting alone§	Persistent wetting¶	p Value
Daytime wetting	1.00 ref.	4.84 (3.11 to 7.54)	–**	10.1 (6.70 to 15.3)	6.98 (4.50 to 10.8)	<0.001
Bedwetting	1.00 ref.	3.83 (2.10 to 6.99)	3.69 (2.21 to 6.17)	0.75 (0.19 to 2.89)	23.5 (15.1 to 36.5)	<0.001
Nocturia	1.00 ref.	1.31 (0.96 to 1.79)	1.23 (0.95 to 1.58)	1.18 (0.81 to 1.73)	2.39 (1.79 to 3.20)	<0.001
Urgency	1.00 ref.	1.32 (0.88 to 1.96)	0.59 (0.38 to 0.90)	1.30 (0.81 to 2.09)	2.10 (1.44 to 3.07)	0.006
Frequent urination	1.00 ref.	1.28 (0.76 to 2.16)	0.78 (0.47 to 1.29)	0.81 (0.38 to 1.71)	1.28 (0.71 to 2.30)	0.84
Low voided volume	1.00 ref.	1.15 (0.74 to 1.79)	0.94 (0.65 to 1.36)	1.28 (0.78 to 2.10)	1.45 (0.92 to 2.27)	0.66
Voiding postponement	1.00 ref.	1.32 (1.03 to 1.71)	0.85 (0.68 to 1.08)	1.94 (1.48 to 2.54)	1.27 (0.95 to 1.70)	0.004
Passing hard stools	1.00 ref.	1.52 (0.90 to 2.58)	0.74 (0.43 to 1.29)	2.17 (1.27 to 3.71)	2.64 (1.63 to 4.27)	0.006
Low stool frequency	1.00 ref.	1.33 (0.99 to 1.80)	0.87 (0.67 to 1.15)	1.39 (0.98 to 1.97)	1.27 (0.90 to 1.78)	0.26

*Normative development of bladder control: comprising normative classes for daytime wetting and bedwetting.

†Delayed attainment of night-time bladder control with daytime wetting: comprising frequent and infrequent delayed bedwetting classes and the non-normative daytime wetting classes.

‡Bedwetting alone (no daytime wetting): comprising all non-normative bedwetting classes and the normative daytime wetting class.

§Daytime wetting alone (no bedwetting): comprising the normative bedwetting class and the non-normative daytime wetting classes.

¶Persistent bedwetting with daytime wetting: comprising the infrequent persistent and frequent persistent bedwetting classes and the non-normative daytime wetting classes.

**The parameter could not be estimated due to insufficient cases.

There were increased odds of daytime wetting at 14 years among those with daytime wetting alone, persistent wetting and delayed attainment of bladder control at 4–9 years, for example, members of the ‘daytime wetting alone’ class in childhood had over a 10-fold increase in the odds of experiencing daytime wetting in adolescence compared to those with normative development of bladder control during childhood.

It is particularly striking that among members of the ‘persistent wetting’ class (persistent bedwetting with daytime wetting) in childhood, there was over a 23-fold increase in the odds of experiencing bedwetting at 14 years, while the odds of bedwetting in adolescence among those with ‘bedwetting alone’ in childhood were substantially lower (around a threefold increase). Children who experienced delayed attainment of bladder control also had increased odds of bedwetting at 14 years.

The odds of nocturia and urgency in adolescence were increased by around twofold in those with persistent wetting. Members of the daytime wetting alone and delayed classes had increased odds of voiding postponement in adolescence.

The odds of passing hard stools in adolescence were increased in those with daytime wetting alone and those with persistent wetting in childhood.

This study found no evidence that the parallel latent classes of daytime wetting and bedwetting were associated with increased odds of frequent urination, low voided volume or low stool frequency in adolescence.

## Discussion

This study describes five distinct trajectories of the development of daytime and night-time bladder control in childhood and finds evidence that they are differentially associated with bladder and bowel symptoms in adolescence. The ‘persistent wetting’ trajectory was more common in boys and was associated with increased odds of bedwetting, daytime wetting, nocturia, urgency and passing hard stools in adolescence. There was a 23-fold increase in the odds of adolescent bedwetting in those with persistent wetting compared with around a threefold increase in those with bedwetting alone. Children with bedwetting alone did not have increased odds of any other bladder or bowel symptoms in adolescence. Daytime wetting alone in childhood was more common in girls and was strongly associated with daytime wetting in adolescence and with voiding postponement and passing hard stools. Children who experienced delayed attainment of bladder control also had increased odds of UI and voiding postponement in adolescence.

The major strength of this study is the availability of repeated measures of bedwetting and daytime wetting across childhood in a large, representative cohort. Using this data, we modelled parallel trajectories of childhood urinary incontinence and related these to adolescent outcomes. Our previous separate trajectory models of daytime wetting and bedwetting did not take into account the comorbidity between these types of incontinence.^21–23^ By refining our earlier models, we have provided further insights into the heterogeneity in the development of continence.

This study has several limitations that need to be considered when interpreting the findings. Adolescent bladder and bowel symptoms were based on self-report rather than clinical diagnosis. It is reassuring that the prevalence and gender distribution of bedwetting and daytime wetting is comparable to other studies of adolescents.^1^ ^6^ ^8^ Using self-reports is more problematic for voiding frequency, voided volume and stool frequency due to recall error and difficulty appreciating what constitutes ‘the norm’. This might explain why we found no evidence for associations with these outcomes. It is possible that 14-year-olds overestimated the bladder and bowel symptoms if they had childhood continence problems. The prevalence of LUTS in our study is, however, comparable to another prospective study of adolescents.^6^ Voiding postponement was the most commonly reported LUTS (almost 14%). To the best of our knowledge, there are no other population-based studies reporting the prevalence of this symptom in adolescents. Postponing voiding is a very common behaviour and is considered problematic only when there is habitual postponement of micturition using holding manoeuvres,^25^ but adolescents in our study were not asked to report this. We were unable to compare the prevalence of the bowel symptoms with other studies because, to the best of our knowledge, there are no epidemiological studies of these symptoms at a similar age. The prevalence of constipation at 0–18 years was reported to be 8.9% in a systematic review, but the prevalence in adolescence was not provided separately.^26^ We identified a class of children with ‘persistent wetting’ (persistent bedwetting with daytime wetting) but, to reduce model complexity, we did not include other daytime LUTS associated with NMNE.^9^ A further limitation is that soiling and constipation were not included in the latent classes. There was no information available on underlying anatomical or neurological causes of incontinence in our sample, but the vast majority of cases of nocturnal enuresis and daytime wetting are known to be functional.^27^ Finally, we did not consider whether treatment for UI might have impacted on the findings. Parents were asked to report whether children had received treatment (bedwetting alarm or medication) for UI at ages 7 and 9 years. Only a small proportion of children (0.2–0.4%) had received treatment and there was no information on the onset or duration of treatment.

This study found that persistent wetting was associated with much higher odds of adolescent bedwetting than bedwetting alone. Cross-sectional studies report that frequent childhood bedwetting tends to be refractory and often accompanied by underlying bladder dysfunction (including daytime wetting)^1^ ^28^ and that bedwetting with OAB symptoms declines more slowly with age than bedwetting without OAB.^29^ Adult UI has been linked to an increased prevalence of UI in childhood,^12–18^ providing evidence that increasing maturity does not guarantee a resolution to these problems. The subtype of persistent and frequent UI has been found to be more common among adults with UI that has persisted since childhood.^12^ ^30^ There is evidence for aetiological differences between children who wet the bed and have daytime symptoms (NMNE)^9^ and children who wet the bed and have no daytime symptoms (MNE).^11^ While bedwetting alone is believed to be a primary problem with nocturnal polyuria, sleep and/or a delay in maturation in the nervous system,^11^ OAB is believed to be involved in the pathophysiology of bedwetting with daytime wetting.^9^ ^31^ The persistent wetting class had increased odds of urgency at 14 years indicating a probable association of OAB syndrome, since urgency is the cardinal symptom.^10^ Persistent wetting in childhood was also associated with increased odds of daytime wetting in adolescence, but the association was not as strong as that found for adolescent bedwetting. Previous studies have shown that daytime wetting decreases from childhood to adolescence.^6^ ^7^ As children mature and develop increasing social and body awareness, adoption of strategies to prevent daytime wetting may result in resolution of the daytime symptoms, but bedwetting may persist. Persistent wetting was also associated with increased odds of nocturia in adolescence (this association persisted after excluding those with any wetting at 14 years—available on request), suggesting that children whose bedwetting remits by adolescence remain at risk of nocturia. A long-term follow-up study found evidence for an association between bedwetting in childhood and nocturia in adolescence and young adulthood (the presence of nocturia was associated with NMNE in childhood and a later age at attainment of nocturnal bladder control).^17^ A clinical study reported that a third of children whose bedwetting was successfully treated with an alarm developed nocturia.^32^ The odds of passing hard stools at 14 years were increased among those with persistent wetting in childhood and with daytime wetting alone. Adolescents with bladder dysfunction often report bowel symptoms and in particular constipation.^8^ ^33^ The retained stool mass in the rectum may compress the bladder leading to bladder contractions (OAB) or to emptying difficulties,^19^ but equally adolescents may drink less to minimise OAB symptoms and frequently contract their pelvic floors in response to the overactive contractions, which may give rise to constipation.

Daytime wetting in childhood was strongly associated with adolescent daytime wetting. Most children with daytime wetting have some form of functional UI with the majority of cases being due to OAB, voiding postponement or dysfunctional voiding (characterised by straining and interrupted stream—not assessed in the current study).^25^ This study found increased odds of voiding postponement in adolescence in those with daytime wetting alone and delayed attainment of bladder control, but not with OAB symptoms. The lack of association with OAB symptoms could be due to the low prevalence of these symptoms or limitations with using self-reports of frequency and volume. Also, ‘daytime wetting alone’ is the smallest class, which could result in a lack of precision in our estimates of the association with the adolescent symptoms. Increased odds of UI in adolescence were also seen in those with delayed attainment of bladder control in childhood. The delayed trajectory comprises children with delayed attainment of night-time bladder control (very low probability of bedwetting by age 9) and any daytime wetting; therefore, it might have been expected that bedwetting would have resolved by adolescence. Delayed development of night-time bladder control has been linked to a genetic disposition and general delay in maturation,^34^ and there is evidence that these children may more susceptible to relapses in bedwetting when exposed to stress.^35^ This could explain why the delayed class has a re-emergence of bedwetting in adolescence.

The awareness of long-term outcomes of childhood incontinence is important in clinical practice, as this implies that some children need to be assessed regularly and prioritised for treatment. Owing to the well-documented economic and social impacts of incontinence, there is a need to identify patterns of childhood incontinence that are less likely to resolve with age. Incontinence becomes harder to treat as children grow older^36^ and more socially unacceptable, leading to significant impacts on quality of life.^37^ Subgrouping of UI is clinically useful because it provides guidance on appropriate interventions^9^ ^38^ and identifies trajectories of childhood incontinence that should receive early intervention. In this study, children with delayed attainment of bladder control, those with daytime wetting alone and those with persistent wetting had increased levels of daytime wetting in adolescence. Although the odds of adolescent daytime wetting were highest among those with childhood daytime wetting alone, there was overlap in the CIs with the other classes. A striking finding of this study is that children with persistent (day and night) wetting had a much higher chance of bedwetting in adolescence than those with delayed attainment of bladder control or bedwetting alone. This finding could imply that particular attention should be paid to persistent bedwetting with accompanying daytime wetting because these children have a high chance of becoming adolescents with UI and other bladder and bowel symptoms. Further research is needed to examine the long-term prognosis of adolescent bladder and bowel symptoms into adulthood and to determine whether similar developmental trajectories can be identified in other prospective cohorts.
