# Supplementary material for: Trajectories of urinary incontinence in childhood and bladder and bowel symptoms in adolescence: prospective cohort study
Source: BMJ Open. 2017 Mar 10;7(3):e014238. doi: 10.1136/bmjopen-2016-014238 (PMC5353296; doi:10.1136/bmjopen-2016-014238)
Supplement: supplementary material [file bmjopen-2016-014238supp.pdf]

Supplementary online material

Statistical analysis used to estimate the parallel trajectory model

This supplementary material presents the methodological framework and procedures we employed to distinguish groups of children with similar developmental trajectories (latent classes) of daytime wetting and bedwetting. We also describe the methods we used to examine the association between the latent classes in childhood and the bladder and bowel symptoms in adolescence.

Earlier work: separate models of daytime wetting and bedwetting

In our previous work, we applied longitudinal latent class analysis (LLCA) separately to daytime wetting[1] and bedwetting[2] data collected from children in the ALSPAC cohort at 4 – 9 years. LLCA is an empirical method, which uses a categorical latent variable to explain the associations observed between a set of binary or categorical repeated measures. The assumption is that patterns in the observed data are due to the presence of unobserved sub-populations (latent classes). The goal is to determine the number of these latent classes and estimate both the relative size of the classes (the class-distribution) and also the developmental pattern exhibited by members of each latent class (the trajectory).

Daytime wetting and bedwetting data were obtained from parental reports when the study children were $4^1/_2$, $5^1/_2$, $6^1/_2$, $7^1/_2$, and $9^1/_2$ years. Parents were asked "How often usually does your child wet the bed?" and were given the options "never"; "less than once a week"; "about once a week"; "2 to 5 times a week"; "nearly every night"; "more than once a night." For daytime wetting, we dichotomised the data to produce a set of binary variables indicating those with daytime wetting (coded 1) or without daytime wetting (coded 0) at each time point. Child A might have responses "10000" for the questions on daytime wetting at 4½, 5½, 6½, 7½, and 9½ years respectively, whereas child B might have responses "11111". Such variability in these patterns of responses is accounted for by a latent factor that groups together children exhibiting similar patterns of development. The result is a number of different latent classes (trajectories). Each class can be seen to exhibit its own trajectory through time by plotting age against the class-specific prevalence of daytime wetting at each time point. Since some children are more easily assigned to a particular class than others, the output from a latent class model is a set of posterior probabilities describing the likelihood of a child being a member of each class. For instance, child A would be likely to have a high probability of being assigned to a group characterized by normal or slightly delayed development, whilst child B would have a higher probability of being a member of a group characterized by persistent daytime wetting.

Due to the greater number of children wetting the bed at each age, we derived a three-category ordinal measure for the bedwetting data: no bedwetting (never: coded 0), bedwetting 'less than once a week' and 'about once a week' (infrequent: coded 1) and bedwetting at least twice a week (i.e. any of the three most frequent response options: coded 2). This third category of frequent bedwetting corresponds to the frequency of bedwetting required for a DSM-V diagnosis of nocturnal enuresis. For bedwetting, child A might have responses "10000" (i.e. a child who has attained nighttime bladder control by age 5½), child B might have responses "21000" (i.e. a child who experiences delayed attainment of nighttime bladder control) and child C might have responses "22221" (i.e. a child with persistent and frequent bedwetting).

The LLCA model assumes that observed heterogeneity in responses is due to a latent (unobserved) grouping in the population. Starting with a single class, additional classes are added until the various assessments of model fit reach an acceptable level. Similar to factor analysis, this latent categorical variable should explain the associations within the set of

repeated measures such that within each latent class, respondents form a single homogeneous group, and furthermore the repeated measurements are independent of each other, i.e. there is local (conditional) independence.
Using LLCA (carried out in Mplus version 7.11), we have previously shown that patterns of daytime wetting at 4 – 9 years could be adequately explained by a four-class solution and patterns of bedwetting could be explained by five-classes.

Daytime wetting classes

(i) Normative development (86.2% of the sample): attainment of daytime bladder control at age 4 – 5 and very low probability of daytime wetting at any age;
(ii) Delayed (6.9%): delayed attainment of daytime bladder control characterised by steadily decreasing prevalence of daytime wetting from 80% at age 4.5 years to 40% at 6.5 years, to less than 10% by 9.5 years;
(iii) Relapsing (3.2%): initial normal development of daytime bladder control, but with an increased probability of daytime wetting at age 6 – 7, followed by a decreased probability up to age 9;
(iv) Persistent (3.7%): relatively high probability of daytime wetting from 4 – 9 years.

Bedwetting classes

(i) Normative: (71.5% of the sample): low probability of bedwetting at any time point;
(ii) Infrequent delayed (14.3%): delayed attainment of nighttime bladder control and decreasing probability of infrequent (<twice a week) bedwetting from 4 – 9 years;
(iii) Infrequent persistent (8.6%): relatively high probability of infrequent bedwetting;
(iv) Frequent delayed (2.4%): high probability of frequent (>= twice a week) bedwetting at age 4 years, which decreased and became more infrequent at 6 – 9 years;
(v) Frequent persistent (3.2%): relatively high probability of bedwetting at least twice a week from 4 – 9 years.

Parallel latent class model of daytime wetting and bedwetting

Deriving *separate* latent class models for daytime wetting and bedwetting ignores the comorbidity between these two continence problems. We derived a "parallel LLCA" in order to describe the repeated bivariate data of daytime wetting and bedwetting in tandem. The purpose of this model was:

(i) To determine the degree of co-morbidity between latent classes of daytime wetting and bedwetting in this population (e.g. to what extent do children who are delayed in attaining daytime bladder control also exhibit delays in attaining nighttime bladder control?) and

(ii) To examine the association between the parallel LLCA and the adolescent outcomes. Figure S1 shows the parallel LLCA model with nocturia at age 14 as an example of an adolescent outcome.

The procedure that we followed to apply the parallel LLCA model to the daytime wetting and bedwetting data consisted of the following stages. First, we started our analyses by deriving separate LLCA models for daytime and nighttime wetting indicators (see above). We then derived the parallel LLCA model including daytime wetting and bedwetting simultaneously.

To determine the optimal number of latent classes of daytime wetting and bedwetting, we estimated parallel LLCA models including all possible combinations of number of classes and analysed each solution with respect to three statistical criteria: Bayesian information criterion (BIC) [3]; entropy[4] and the analysis of the standardized residuals and overall bivariate Pearson chi square statistics associated with them. It is rare that all indicators of model fit point to the same solution. The accepted approach is to evaluate the statistical evidence alongside face validity, resemblance to other results in the literature, and pragmatic issues such as class size.
The statistics summarizing the overall model performance are shown in Table S1. The residuals for the solution with 5 classes on the bedwetting dimensions were all within a reasonably narrow band (only few of the residuals were outside the +/-2.0 limits) (Figure S2). Other solutions with a fewer number of bedwetting classes had residuals that were more dispersed. The overall summary statistics showed that the best model in terms of fit for daytime wetting is the one with 4 latent classes. Thus, the final parallel LLCA solution that we selected had 4 classes for daytime wetting and 5 classes for bedwetting. The four-by-five class solution corresponding to each combination of daytime wetting and bedwetting had a good fit and adequately explained the longitudinal heterogeneity in development of both daytime and nighttime bladder control. Figures S3 and S4 show the prevalence of the latent classes of daytime wetting and bedwetting and their developmental trajectories over time. The four-by-five class solution represented 20 separate subgroups corresponding to each combination of daytime wetting and bedwetting. It is not practical to examine the risk of each adolescent outcome within each of these groups. Consequently we collapsed these groups into five distinct, clinically relevant classes.

Statistical analysis to examine the association between the parallel latent classes and the adolescent bladder and bowel symptoms

We examined the association between the latent classes and the adolescent outcomes using a series of logistic regression models considering each binary outcome in turn with the parallel latent class model as the exposure. Logistic regression models were estimated in Latent Gold (version 5.0). We obtained parameter estimates using the "Modal ML" 3-step method[5] and since implemented in Mplus with the "auxiliary (r3step)" command. This enabled outcome data to be modelled whilst avoiding any distortion of the latent class solution. In step one, the latent class model is estimated using an unconditional LLCA (i.e. a model in the absence of covariates). This model is used to derive class-assignment probabilities i.e. the probability with which each respondent is believed to belong to each class. Respondents are then assigned to the class for which their probability is greatest creating a non-latent classification (step two). Finally in step 3, measurement error inherent in the non-latent classification is quantified and used to reproduce latent classes using a set of logit constraints. This approach has been shown to produce less-biased estimates than traditional three-step methods such as standard probability weighting or modal assignment, whilst avoiding the problem of covariates impacting on the measurement model itself [6].

Impact of missing data on the results of the analysis investigating associations between the latent classes of daytime wetting and bedwetting at 4 – 9 years and bladder and bowel symptoms at 14 years.

To conduct this diagnosis we first created dummy variables for each outcome variable in which 1 indicated missing data and 0 indicated observed data. We then exported data to Mplus and predicted missing values using class memberships as an exposure (nominal variable with 5 categories). To avoid problems with bias we applied the D-matrix approach.

The differences in probability of missing values were tested by means of Wald tests with the null hypothesis stating that the probability of missingness is the same across all categories of exposure variable. The results of those regressions (in probability scale) are displayed in Table S2. The results did not reveal any major differences in missing values across latent classes extracted. Wald statistics obtained for every model confirm this conclusion by showing that there are no statistically significant differences in proportions of missing data across extracted latent classes. This result suggests that the missing data mechanism was completely at random (MCAR). Thus, the impact of missing values on the results seemed to be minimal and therefore we decided that there was not a strong argument for employment of multiple imputation methods to impute missing data.

References

1. Heron J, Joinson C, Croudace T, von Gontard A. Trajectories of daytime wetting and soiling in a United Kingdom 4 to 9-year-old population birth cohort study. *Journal of Urology*. 2008;179:1970-1975.
2. Sullivan S, Joinson C, Heron J. Factors predicting atypical development of nighttime bladder control: a prospective cohort study. *J Dev Behav Pediatr*. 2015;36(9):724-33.
3. Schwarz G. Estimating the dimension of a model. *Ann Stat*. 1978;6:461–464.
4. McCutcheon AL. *Latent Class Analysis* (Sage University Paper series on Quantitative Applications in Social Sciences, No. 07-064). Newbury Park, CA: Sage; 1987
5. Vermunt, J. K. Latent Class Modeling with Covariates: Two Improved Three-Step Approaches. *Political Analysis*. 2010;18, 450–469.
6. Asparouhov T, Muthen BO. *Auxiliary Variables in Mixture Modeling: 3-Step Approaches Using Mplus*. Mplus Web Notes: No 15. 2013. Available at: http://www.statmodel.com/examples/webnotes/webnote15.pdf. Accessed February 30, 2015.
