# Supplementary material for: Trajectories of urinary incontinence in childhood and bladder and bowel symptoms in adolescence: prospective cohort study
Source: BMJ Open. 2017 Mar 10;7(3):e014238. doi: 10.1136/bmjopen-2016-014238 (PMC5353296; doi:10.1136/bmjopen-2016-014238)

Table S1. Fit statistics of the parallel LLCA model (n = 8,751)

Number of Classes for Daytime Wetting (DW) and Bedwetting (BW)	BIC[1]	Entropy[2]	Overall bivariate Pearson Chi-square[3]
DW: 3 BW: 3	55,283	0.824	725.0
DW: 3 BW: 4	54,935	0.799	404.1
DW: 3 BW: 5	54,801	0.805	208.3
DW: 4 BW: 3	55,237	0.841	680.5
DW: 4 BW: 4	54,882	0.817	345.7
DW: 4 BW: 5	54,756	0.822	149.4
DW: 5 BW: 3	55,259	0.831	673.2
DW: 5 BW: 4	54,909	0.809	337.5
DW: 5 BW: 5	54,801	0.810	135.5

1. BIC (Bayesian information criterion) is the traditional fit statistic for comparing mixture models and will typically decrease and then increase after the incremental additional of classes. Using this statistic, the model with the lowest BIC would be deemed optimal.

2. Entropy is a measure of classification accuracy, and while it is generally of little use in determining the optimal model, it indicates the level of bias that one would expect were a standard 3-step estimation to be performed.

3. Bivariate model fit information provides an assessment of conditional independence. Conditional independence is an assessment of the remaining association between each pair of measurements once heterogeneity accounted for when a latent class has been removed. There is currently no accepted threshold for this measure; it is common to observe marked improvements (reductions) followed by smaller changes.

Table S2. Probability of missing data on the bladder and bowel symptoms at 14 years cross each latent class

	Bedwetting alone		Daytime wetting alone		Persistent wetting		Delayed		Normative		Wald	df	p
Missing daytime wetting data at 14 years	probability	S.E.	probability	S.E.	probability	S.E.	probability	S.E.	probability	S.E.			
0 [no missing]	0.647	0.018	0.713	0.033	0.618	0.025	0.675	0.027	0.666	0.007	7.56	4	0.1
1 [missing]	0.353	0.018	0.287	0.033	0.382	0.025	0.325	0.027	0.334	0.007			
Missing bedwetting data at 14 years													
0 [no missing]	0.646	0.018	0.717	0.033	0.622	0.025	0.677	0.027	0.665	0.007	7.46	4	0.1
1 [missing]	0.354	0.018	0.283	0.033	0.378	0.025	0.323	0.027	0.335	0.007			
Missing urgency data 14 years													
0 [no missing]	0.652	0.018	0.722	0.033	0.620	0.025	0.672	0.027	0.665	0.007	7.41	4	0.1
1 [missing]	0.348	0.018	0.278	0.033	0.380	0.025	0.328	0.027	0.335	0.007			
Missing frequent urination data at 14 years													
0 [no missing]	0.648	0.018	0.722	0.033	0.616	0.025	0.671	0.027	0.663	0.007	8.23	4	0.0
1 [missing]	0.352	0.018	0.278	0.033	0.384	0.025	0.329	0.027	0.337	0.007			
Missing low voided volume data at 14 years													
0 [no missing]	0.646	0.018	0.718	0.033	0.620	0.025	0.675	0.027	0.659	0.007	6.98	4	0.1
1 [missing]	0.354	0.018	0.282	0.033	0.380	0.025	0.325	0.027	0.341	0.007			
Missing voiding postponement data at 14 years													
0 [no missing]	0.644	0.018	0.717	0.033	0.622	0.025	0.679	0.026	0.662	0.007	7.36	4	0.1
1 [missing]	0.356	0.018	0.283	0.033	0.378	0.025	0.321	0.026	0.338	0.007			
Missing hard stools data at 14 years													

0 [no missing]	0.643	0.018	0.714	0.033	0.623	0.025	0.676	0.027	0.660	0.007	6.65	4	0.1
1 [missing]	0.357	0.018	0.286	0.033	0.377	0.025	0.324	0.027	0.340	0.007			
Missing stool frequency data at 14 years													
0 [no missing]	0.639	0.018	0.711	0.033	0.620	0.025	0.668	0.027	0.650	0.007	6.06	4	0.1
1 [missing]	0.361	0.018	0.289	0.033	0.380	0.025	0.332	0.027	0.350	0.007			
Missings nocturia data at 14yo													
0 [no missing]	0.643	0.018	0.721	0.033	0.620	0.025	0.679	0.026	0.661	0.007	8.05	4	0.0
1 [missing]	0.357	0.018	0.279	0.033	0.380	0.025	0.321	0.026	0.339	0.007			

Figure S1. Example parallel model with nocturia at age 14 as the adolescent outcome

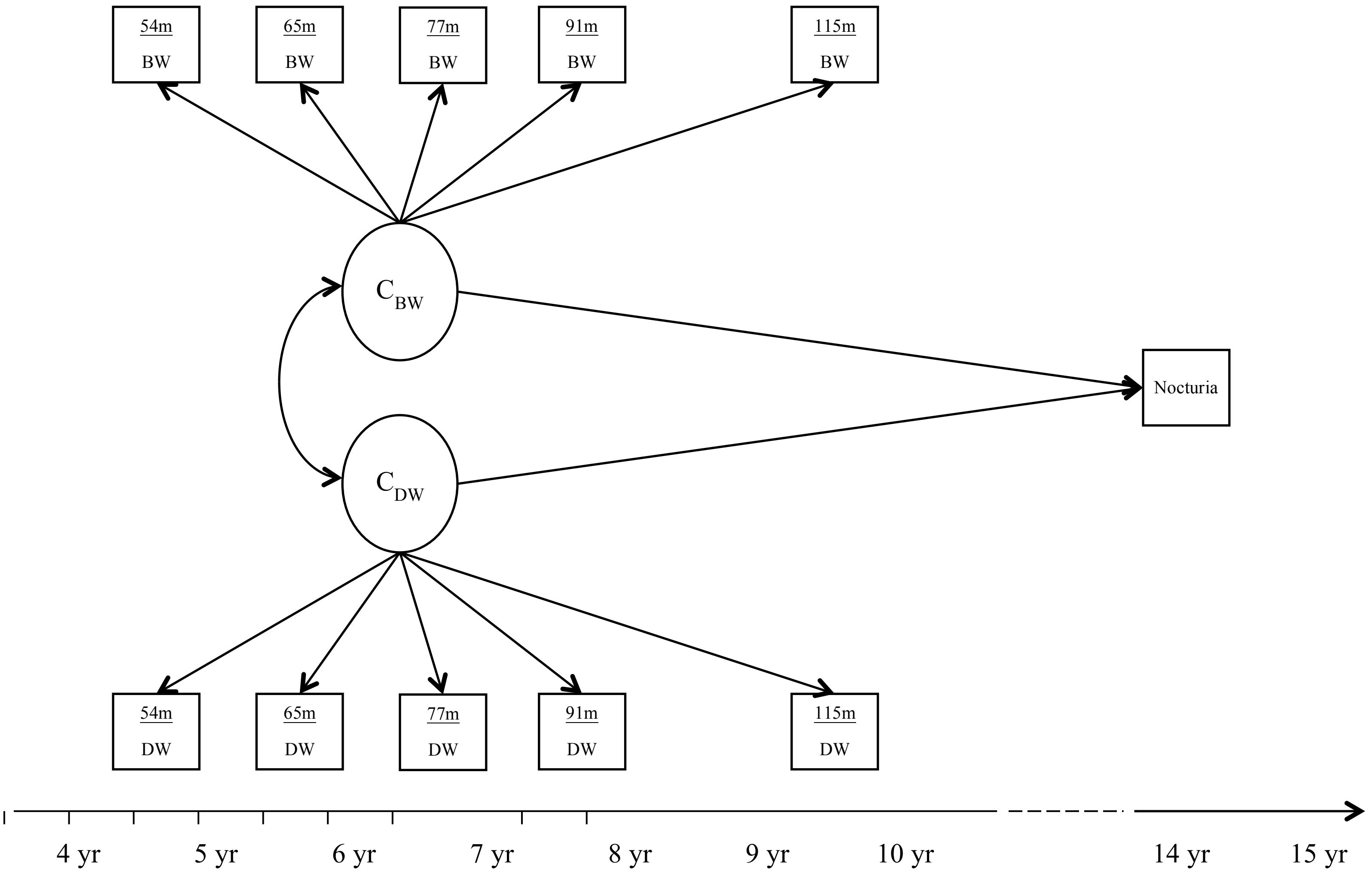

BW: Bedwetting; DW: Daytime Wetting.; C_{BW} and C_{DW} represent latent classes for bedwetting and daytime wetting respectively.

Figure S2. Model bivariate residuals

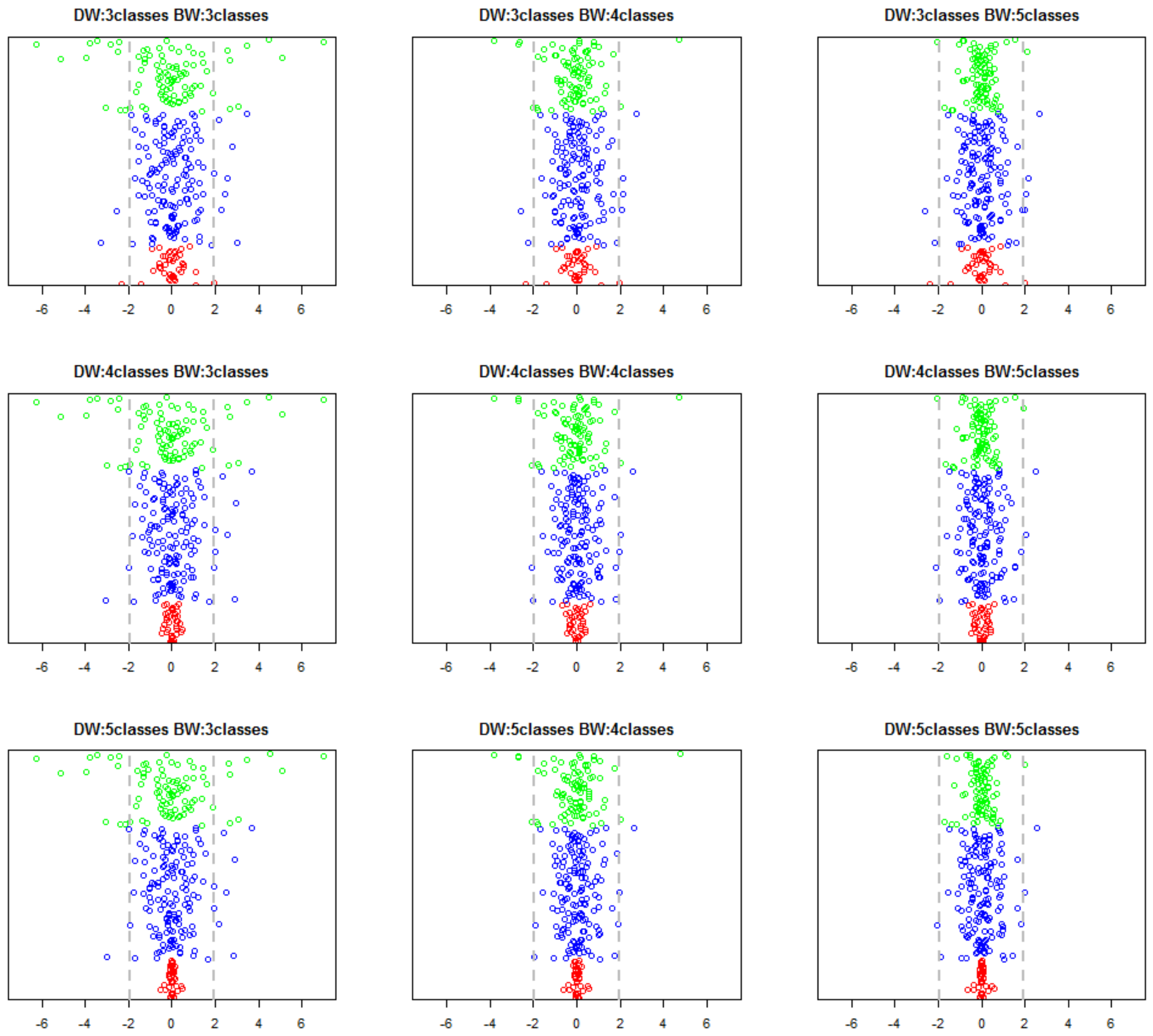

Figure S3. Parallel LLCA model: prevalence of daytime wetting latent classes and their developmental trajectories over time

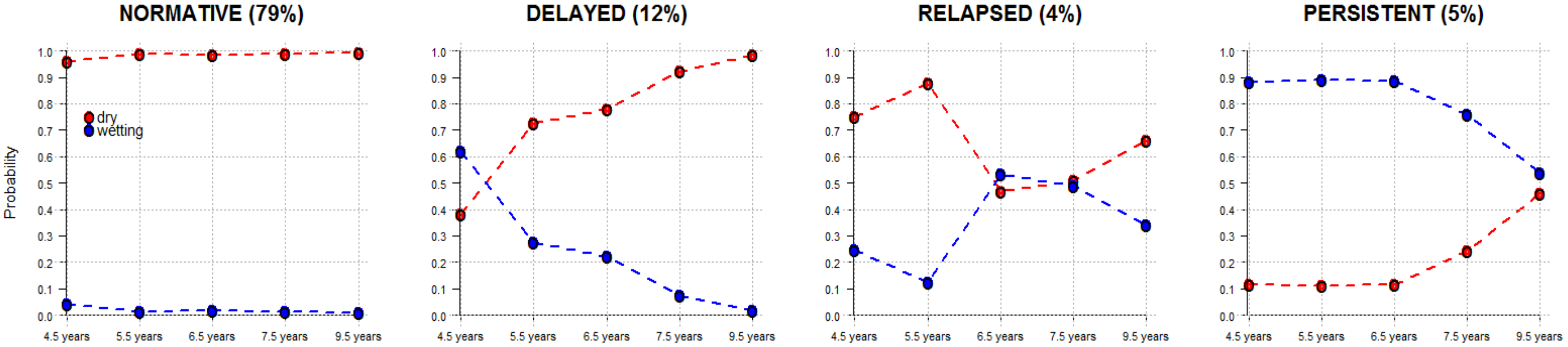

Figure S4. Parallel LLCA model: prevalence of bedwetting latent classes and their developmental trajectories over time

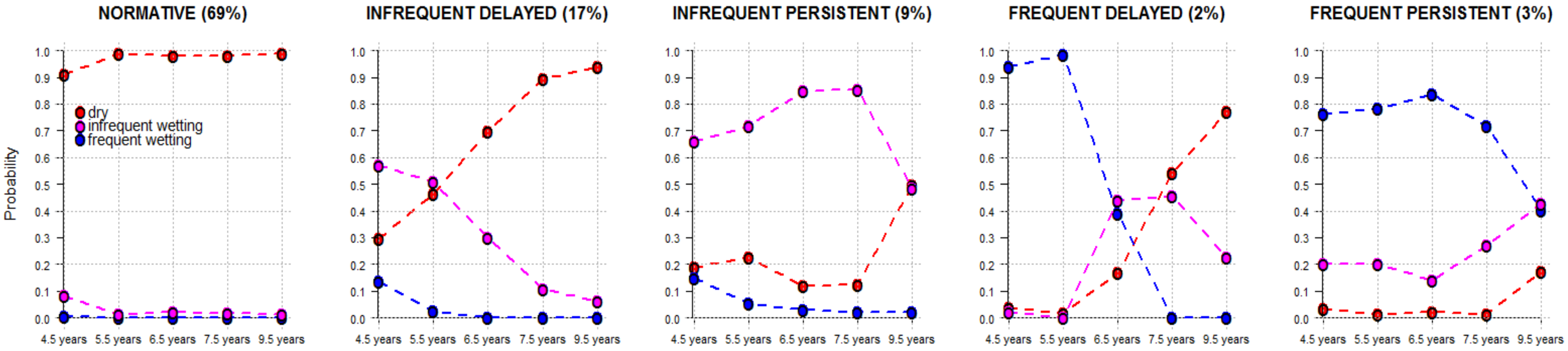

Supplement: supplementary tables and figures [file bmjopen-2016-014238supp_tables_and_figures.pdf]
